# Ewing Sarcoma of the Posterior Fossa in an Adolescent Girl

**DOI:** 10.1155/2014/439830

**Published:** 2014-12-29

**Authors:** Andreas M. Stark, Ivo Leuschner, H. Maximilian Mehdorn, Alexander Claviez

**Affiliations:** ^1^Department of Neurosurgery, Schleswig-Holstein Medical University in Kiel, Arnold-Heller Strasse 3, Haus 41, 24105 Kiel, Germany; ^2^Institute of Pathology, Children's Tumor Registry, Schleswig-Holstein Medical University in Kiel, Arnold-Heller Strasse 3, Haus 14, 24105 Kiel, Germany; ^3^Department of Pediatrics, Schleswig-Holstein Medical University in Kiel, Arnold-Heller Strasse 3, Haus 9, 24105 Kiel, Germany

## Abstract

Medulloblastoma, astrocytoma, and ependymoma represent the most common infratentorial tumors in childhood, while Ewing sarcomas in that localization are extremely rare. A large left infratentorial space-occupying lesion was diagnosed in a 12-year-old girl with signs of increased intracranial pressure. Following total tumor resection, histological and molecular examination revealed Ewing sarcoma with rearranged *EWSR-1* gene. The patient achieved complete remission following adjuvant chemotherapy and radiotherapy according to Euro-EWING 2008 treatment protocol. Intracranial Ewing sarcoma, although rare, should be an important differential diagnosis of intracranial tumors in childhood which requires aggressive multimodal treatment.

## 1. Introduction

Intracranial tumors represent the most common solid neoplastic lesions in children. Their incidence is in the range of 2–4 per 100.000 per year [1]. Up to 70% are located in the posterior fossa. The most common histological types are astrocytoma, medulloblastoma, and ependymoma [2, 3] whereas plexus papilloma, schwannoma, hemangiopericytoma atypical rhabdoid tumors, brainstem glioma, and meningioma represent uncommon tumors [4, 5]. Here we report an adolescent presenting with a tumor of the Ewing sarcoma tumor family (ESTF) presenting with clinical signs of intracranial pressure. Ewing sarcomas represent the second common malignant bone tumors in childhood and adolescence and are most commonly found in the pelvis, femora, and ribs but may affect any bone and are extremely rare in the posterior fossa [6].

## 2. Case Report

**Figure 1:**
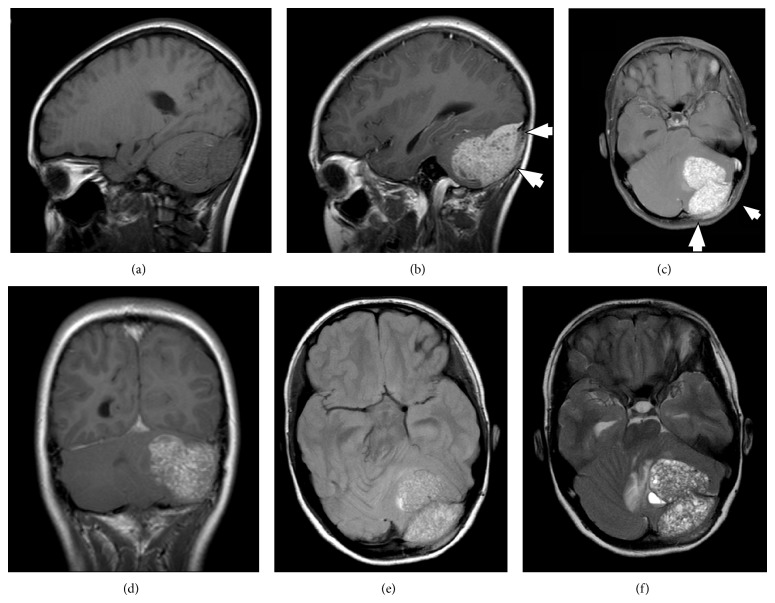
Preoperative MRI demonstrates a large contrast-enhancing dural-based tumor extending into the left posterior fossa forming two bulks. (a) T1-weighted noncontrast sagittal MRI. (b) T1-weighted contrast-enhanced sagittal view: short arrows indicate bone erosion. (c) T1-weighted contrast-enhanced axial MRI: short arrows indicate bone erosion. (d) T1-weighted contrast-enhanced coronary MRI. (e) Fluid attenuated inversion recovery- (FLAIR-) weighted coronal MRI. (f) T2-weighted axial view.

A 12-year-old girl was admitted with a several-weeks history of headache, vomiting, and abdominal pain. Neurological examination revealed slight ataxia but was otherwise normal. The past medical history as well as the family history was uneventful. Magnetic resonance imaging (MRI) of the brain revealed a large space-occupying lesion of the left posterior fossa measuring 5.4 × 5 × 3.9 cm. The lesion was based on the convexity of the left infratentorial space and extension into the posterior fossa with bone erosion extending towards the left occipital space. The intracranial tumor part consisted of two parts, enhanced gadolinium, and was accompanied by peritumoral edema mainly focused on the vermis and the contralateral white matter and dislocating the transverse sinus (Figure 1).

The patient underwent total tumor resection via a left-sided suboccipital approach. First, an extracranial tumor layer overlying the left posterior fossa and infiltrating the muscle tissue was removed. The infiltrated bone segment extended from the posterior fossa to the left occipital region covering the occipital pole. All affected bone parts were removed. Then, an epidural tumor layer overlying the transverse sinus was removed. After decompressing the dura the transverse sinus was found to be intact. Intradural inspection at this site revealed no residual tumor. Then, the infratentorial intradural tumor was excised completely including the dura. The tumor was firm with only few small nutrition vessels originating from the leptomeninges. After tumor removal, the dural defects were covered with sutured Neuropatch. The resected bone was replaced with methyl-methacrylate pasty.

**Figure 2:**
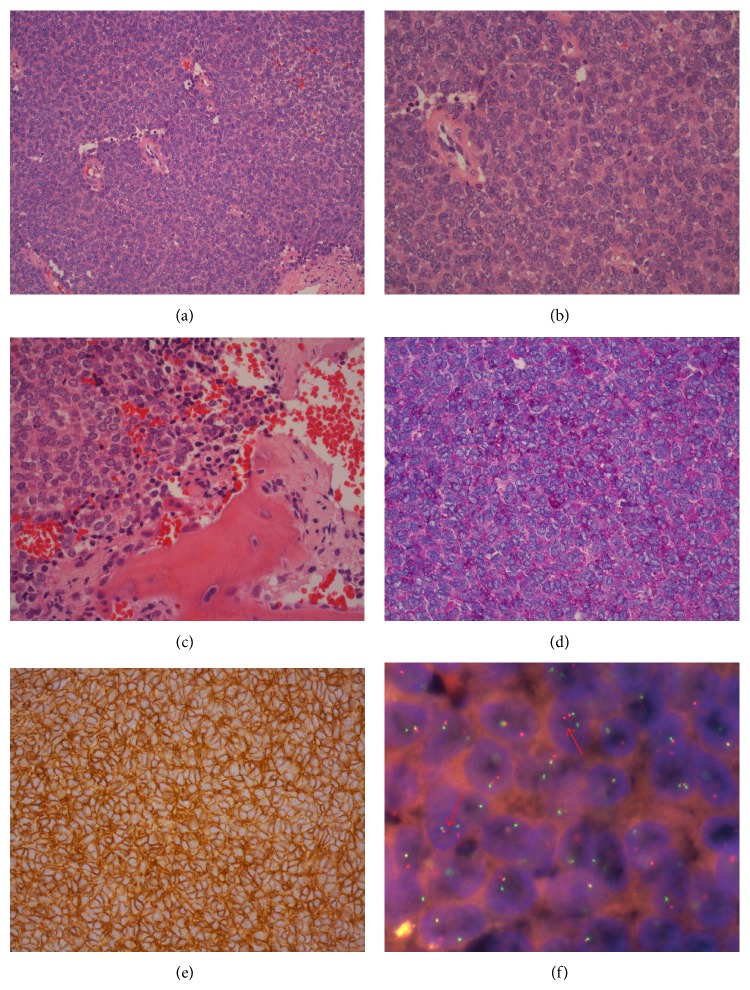
(a) Diffuse sheets of tumor cells. H&E, ×20. (b, c) Tumor cells with scanty cytoplasm and round to ovoid nuclei infiltrating the bone. H&E, ×40. (d) High amount of glycogen in the tumor cells by granular PAS positivity. PAS, ×40. (e) Membraneous expression of neuronal marker CD99. ABC, ×40. (f) Split signal of* EWRS1* gene located in the tumor cells (arrows). FISH technique, ×100.

Histologically, the tumor consisted of small to ovoid cells with scanty cytoplasm. Nuclei were round and hyperchromatic. The cells were arranged in large sheets (Figures 2(a) and 2(b)) with focal necrosis. Tumor cells infiltrated and destructed the bone trabeculae in the periphery (Figure 2(c)). PAS staining revealed a granular positivity (Figure 2(d)). Reticulum staining showed that almost no proliferating fibers were found within the tumor. Tumor cells were positive for CD99 (Figure 2(e)) but negative for myogenin, synaptophysin, CD3, CD79a, cytokeratins, and S100. Molecular analysis demonstrated a break in the* EWSR-1* gene on chromosome 22q12 using FISH (fluorescence in situ hybridization) technique (Figure 2(f), arrows) consistent with a diagnosis of Ewing sarcoma.

Postoperatively, bone scintigraphy, computed tomography of the thorax, and abdomen sonography revealed no signs of distant metastases. The patient was included into the prospective multinational treatment protocol Euro-EWING 2008 (EudraCT number: 2008-003658-13). Treatment consisted of six cycles of VIDE chemotherapy (vincristine, ifosfamide, doxorubicin, and etoposide) [7], followed by conventional radiotherapy (45 Gy) with safety margins including the initial tumor bed, scars, and site of postoperative drain and eight cycles of consolidation chemotherapy with VAC (vincristine, actinomycin D, and cyclophosphamide). During radiotherapy actinomycin D was omitted. The patient was randomized to receive nine add-on cycles of zoledronic acid. Twenty-five months after tumor resection, 13 months after completion of chemotherapy, and eight months after the end of add-on therapy with zoledronic acid, the girl remained in complete clinical remission without neurological deficits.

## 3. Discussion

Ewing sarcoma belongs to the group of highly malignant small blue and round cell neoplasms. They most commonly occur in children and young adults [8, 9]. Although any human bony structures can be involved, the tumor is predominantly located in pelvis, large hollow bones, and ribs. Primary intracranial location is extremely rare [10]. Advances in diagnosis and treatment have resulted in improved outcome [9]. The mainstay of local treatment is surgery with or without radiotherapy embedded into neoadjuvant chemotherapy and following local treatment additional chemotherapy to eliminate residual tumor cells [9]. Recently, tumor-specific genetic alterations have gained interest [8, 11]. Mutations in the* EWSR-1* gene (Ewing sarcoma breakpoint region 1, 22q12.2) are known to cause ESFT as well as other mesenchymal tumors. The* EWSR-1* gene region encodes for a multifunctional protein involved in multiple cellular processes including gene expression and cell signaling [12]. Rearrangements within the* EWSR-1* gene are characteristic for tumors of ESFT [13].

Limited data exists concerning the intracranial location of Ewing sarcoma. To our knowledge, only 39 cases have been published so far, plus our own case [13, 14]. Data suggest that primary intracranial Ewing sarcoma affecting children typically presents as circumscribed, contrast-enhancing, dura-based extra-axial mass [10, 13, 15]. Most cases seem to arise from the convexity of the skull [14]. Our case supports this observation. Tentorial ESFT, however, has also been reported in a child [15] showing intratumoral hemorrhage at diagnosis. Two additional cases of “primary extraosseous Ewing sarcoma” have been reported to arise “near” the meningeal structures [6].

Despite their highly malignant nature, intracranial Ewing sarcomas have a fair chance of cure. Five-year survival rates are in the range of 60% given radical treatment with maximum surgical removal, chemotherapy, and radiotherapy [16].

In conclusion, intracranial Ewing sarcoma is a very rare manifestation of this aggressive tumor. Treatment should be performed within prospective multicentric clinical trials and consists, if possible, of complete tumor resection and intensive chemotherapy and radiotherapy.

## Supplementary Material

Postoperative MRI findings in sagittal and axial views obtained at day 1 following tumor resection and 2 years later during follow-up showing no residual tumor manifestation are provided as Supplementary Material in the online version of the Journal.
