# Supplementary figures and images for: Ewing Sarcoma of the Posterior Fossa in an Adolescent Girl
Source: Case Rep Med. 2014 Dec 29;2014:439830. doi: 10.1155/2014/439830 (PMC4295441; doi:10.1155/2014/439830)

R A F

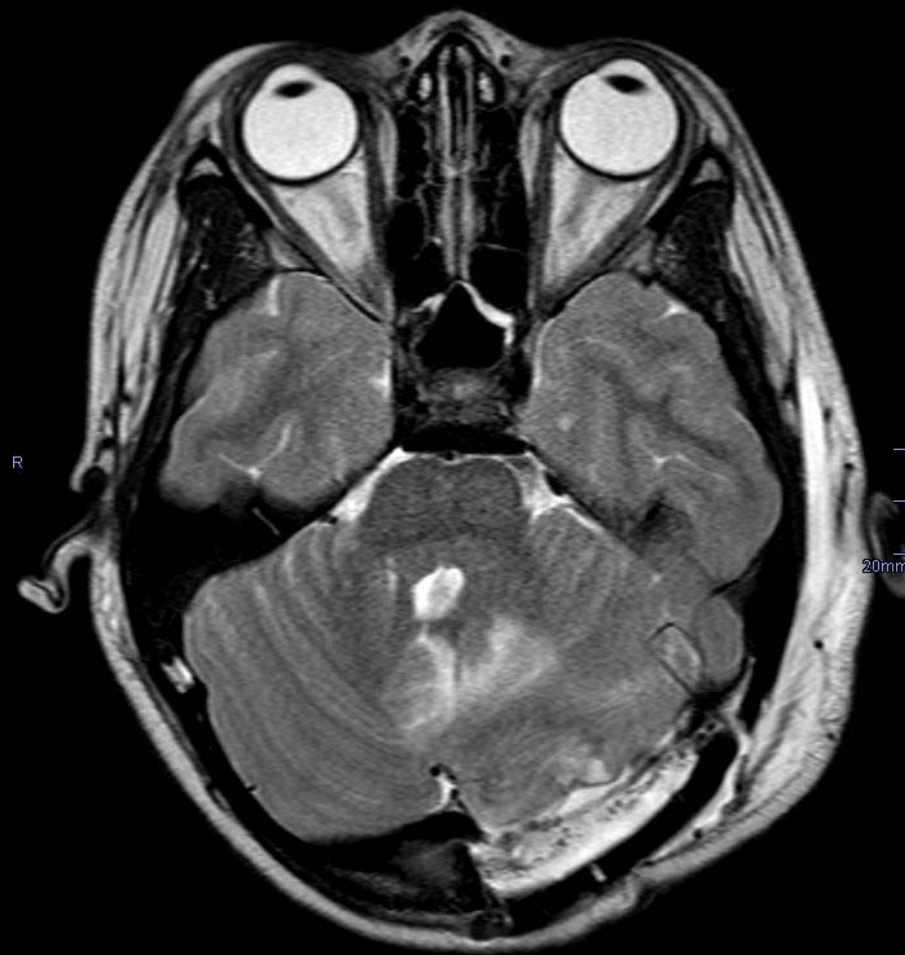

A H

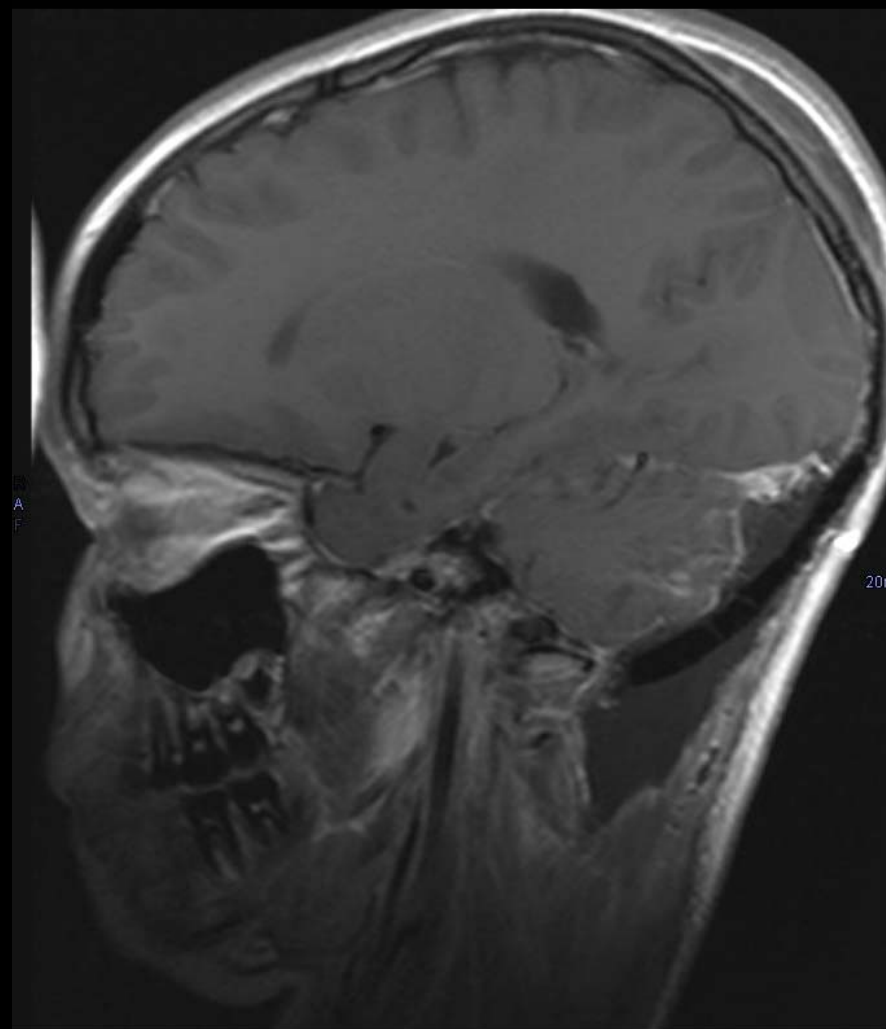

day 1 post operation

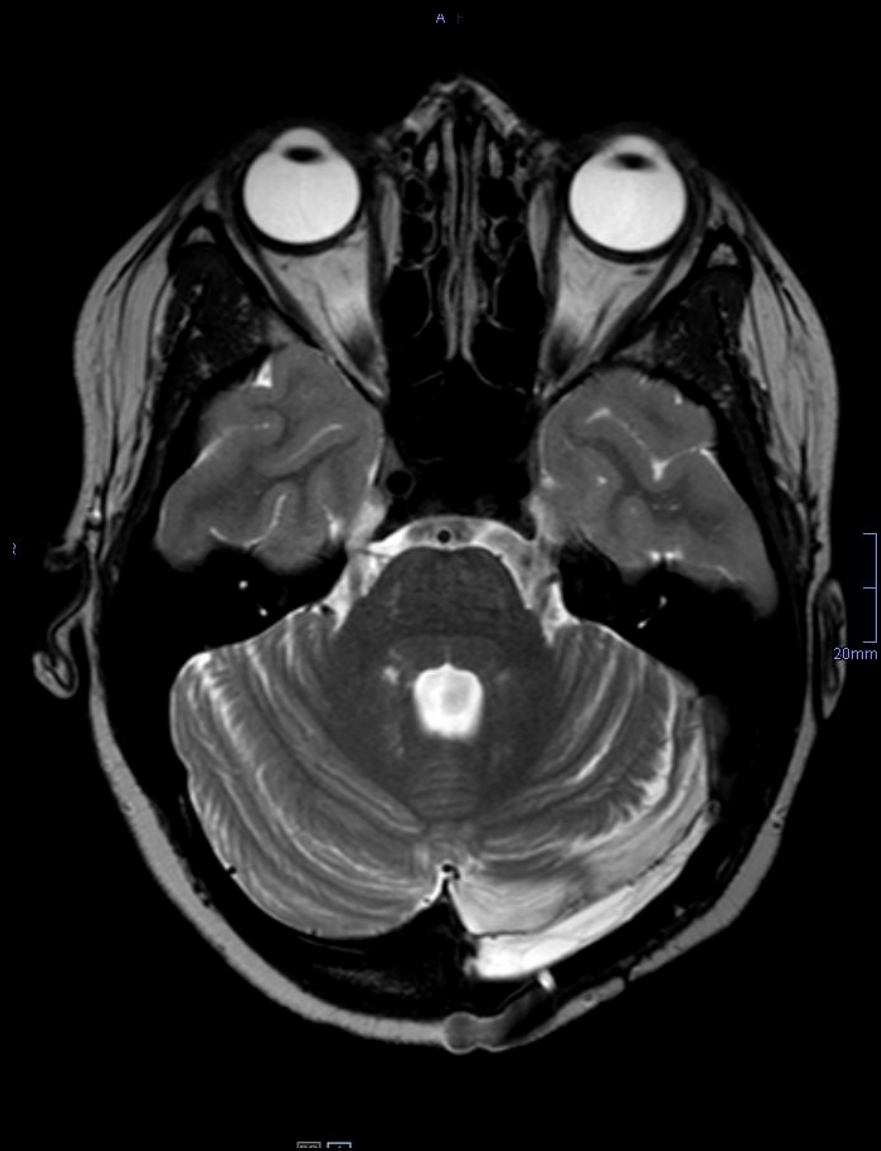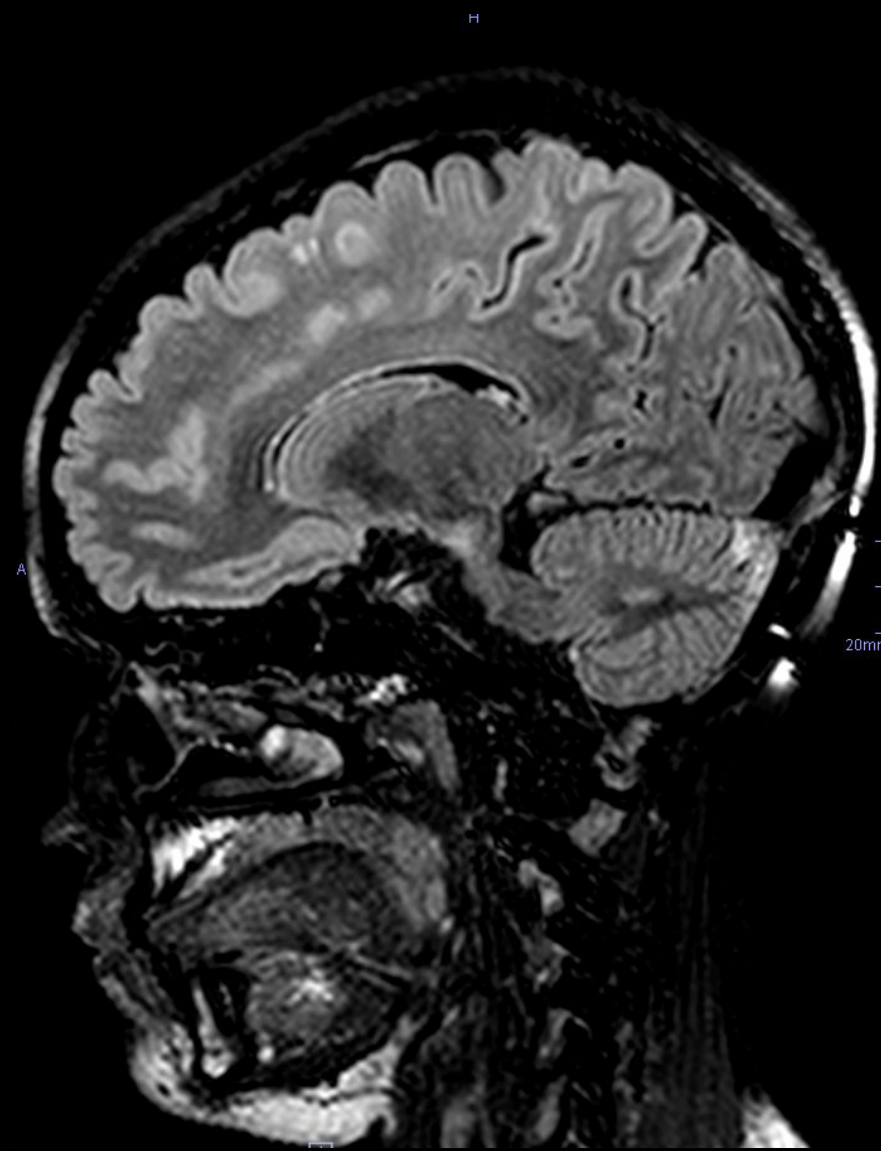

2 years postoperatively

Supplement: Supplementary file 1 — Postoperative MRI findings in sagittal and axial views obtained at day 1 following tumor resection and 2 years later during follow-up showing no residual tumor manifestation are provided as Supplementary Material in the online version of the Journal. [file 439830.f1.pdf]
